# Foot shape is related to load-induced shape deformations, but neither are good predictors of plantar soft tissue stiffness

**DOI:** 10.1098/rsif.2022.0758

**Published:** 2023-01-18

**Authors:** Robert W. Schuster, Andrew G. Cresswell, Luke A. Kelly

**Affiliations:** School of Human Movement & Nutrition Sciences, The University of Queensland, Brisbane, Australia

**Keywords:** three-dimensional scanning, foot biomechanics, longitudinal arch, plantar fascia, statistical shape modelling, transverse arch

## Abstract

Modern human feet are considered unique among primates in their capacity to transmit propulsive forces and re-use elastic energy. Considered central to both these capabilities are their arched configuration and the plantar aponeurosis (PA). However, recent evidence has shown that their interactions are not as simple as proposed by the theoretical and mechanical models that established their significance. Using three-dimensional foot scans and statistical shape and deformation modelling, we show that the shape of the longitudinal and transverse arches varies widely among the healthy adult population, and that the former is subject to load-induced arch flattening, whereas the latter is not. However, longitudinal arch shape and flattening are only one of the various foot shape–deformation relationships. PA stiffness was also found to vary widely. Yet only a small amount of this variability (approx. 10–18%) was explained by variations in foot shape, deformation and their combination. These findings add to the mounting evidence showing that foot mechanics are complex and cannot be accurately represented by simple models. Especially the interactions between longitudinal arch and PA appear to be far less constrained than originally proposed, most likely due to the many degrees of freedom provided by the structural complexity of our feet.

## 1. Introduction

As the only habitual biped among the primates, humans are not only unique in how we move about but also in our anatomy. The anatomical adaptation for bipedal gait is reflected in our feet, which display distinctive structural features and mechanical properties. With its robust calcaneus, short toes, permanently adducted hallux and pronounced longitudinal and transverse arches (LA and TA, respectively), the human foot is said to be well adapted for maintaining balance, absorbing impact energy, transmitting propulsive forces to the ground and recycling stored elastic energy [1]. Specifically, key structural features, the LA and the plantar aponeurosis (PA—which spans the length of the LA), are thought to be involved in both force transmission and energy recycling. The LA by itself is thought to provide the foot with a degree of passive stiffness, with arch height proposed as an important determinant of this stiffness [2–4].

Despite the differences in the appearance of our feet and those of our closest evolutionary relatives, there is also considerable variation in foot shape across our species. The LA in particular is frequently recognized as a defining feature between humans and primates [1,5–8], yet has also been identified as a major source of shape variation among humans, with substantial efforts placed into quantifying the variations in arch shape and height within and between different human populations [2,9–13]. However, these efforts have commonly decimated the complex external shape of the foot through the use of one- or two-dimensional measures. Nevertheless, the recent application of three-dimensional morphometrics to analyse foot shape [14–16] has confirmed that the LA is one of the areas of greatest shape variation within the foot while also documenting shape variations across other regions of the foot that occur with the same frequency and intensity. Considering how widely LA shape varies across healthy populations capable of walking and running (including completely flat feet) and that LA height has also been shown to be a determinant of its stiffness [2–4], the question arises whether and to what extent a stiff LA is a necessary requirement for modern human gait?

One way in which our feet may generate stiffness is the windlass mechanism, which proposes that the PA raises the LA by pulling its endpoints (the ball and heel) together as the toes dorsiflex. Based on evidence from cadaveric feet and elaborate mechanical models [17], this windlass mechanism was originally thought to help stiffen the foot by counteracting LA compression during push-off. However, closer inspection using biplanar videoradiography data obtained during walking and running [18,19] has shown considerable mobility (rather than rigidity) of the LA during push-off. In fact, contrary to the classic understanding of engaging the windlass mechanism to raise the LA and stiffen the foot, Welte *et al*. [20] recently reported that during static loading tasks the windlass mechanism actually makes the arch less stiff, as a higher LA when the windlass is engaged seems to facilitate a greater range of motion for movement. Another recent investigation by Davis & Challis [21] showed that increasing tension in the PA through increased metatarsophalangeal (MTP) joint dorsiflexion does in fact increase LA stiffness during walking, but that foot stiffness can also be increased without increasing PA tension. More importantly, this study also showed that increased LA stiffness may not necessarily favour the energetic efficiency of walking. Venkadesan *et al*. [22] cast further doubt on the importance of the windlass mechanism in stiffening the foot by proposing the TA as another major source of longitudinal stiffness for the foot. Nonetheless, without information on the variability of TA curvature across the healthy human population, it is not yet clear whether the reported wide spread in midfoot mobility during walking [23,24] is related to differences in LA or TA curvature, or a combination of the two. Furthermore, there is evidence that the differences in midfoot motion between low- and high-arched feet while in contact with the ground when walking is multi-dimensional and not limited to just the sagittal plane [25]. Together, these findings raise the question of whether LA compression is just one of many relevant factors that modulate foot stiffness, and one which may vary widely among humans, despite potentially overlapping foot shape characteristics.

The idea of a mobile rather than rigid LA being of benefit is not novel. In fact, it is a requirement for the arch-spring mechanism, which proposes that the LA compresses and recoils with each step while storing and releasing elastic energy in the PA [26]. The arch-spring mechanism has been shown to have an important influence on the energetic cost of running and is therefore thought to be another defining function of the modern human foot [26–28]. Unfortunately, there is no information on the effects of varying LA shape and stiffness on the arch-spring mechanism. These can only be inferred from studies investigating the effect of artificially restricting LA compression [27,29] as well as similar spring-like mechanisms in flat-footed monkeys [30,31]. The former studies showed that restricting LA compression results in an increased metabolic cost of running from the resulting reduction in PA strain. Yet, reduced LA compression could be expected from a high, stiff LA [2,4,32,33] but also a low LA with less potential for compression. Meanwhile, the latter studies showed that pig-tailed macaque, vervet monkey and gibbon feet, completely devoid of a LA, are also capable of storing and releasing elastic energy in their plantar tendons and ligaments by employing a ‘reversed arch’ mechanism [30,31]. While there is no evidence of this mechanism being employed by humans with flat feet, it demonstrates that there is more than one way of achieving the same outcome, that is, enhanced locomotor economy. Therefore, differently shaped feet might not only benefit from different interactions between LA and PA, but also from more or less compliant PAs.

Though the windlass and arch-spring mechanisms assume different behaviours from the PA (rigid rope versus compliant spring, respectively), Welte *et al.* [19] showed that the PA manages to act both as a compliant spring that elongates and recoils, as well as a rigid rope that raises the LA throughout different portions of the stance phase during running. These different behaviours are driven by the compression and subsequent recoil of the LA and simultaneous toe dorsiflexion, showing once again how closely LA and PA interact. The interaction between LA and PA is also reflected in less complex static conditions, during which the PA is thought to help maintain the integrity of the arched configuration of the foot, even in the absence of muscles [34–36], with its thickness being identified as a key predictor of foot posture [37]. Yet, despite its importance for both static and dynamic foot mechanics, *ex vivo* data from 12 specimens has indicated that similar to the vast variation in foot shapes, there seems to be considerable inter-subject variation in PA stiffness [38]. Nonetheless, both the extent of variability of *in vivo* PA stiffness across the healthy population and how this affects foot shape and deformation remain to be determined.

Considering the ongoing debate regarding the relationship between the shape of the LA, TA and the stiffness of our feet, this investigation aims to determine whether the shape of a foot is related to the deformations it undergoes when subjected to load. Furthermore, since the PA is suggested to be a determining component of LA shape and stiffness, this investigation will also explore the natural variations in PA stiffness, and whether they are related to foot shape and deformation.

## 2. Methods

### 2.1. Participants

Ninety-one healthy adults, between the ages of 18 and 40 years (46 female, 45 male; 26 ± 6 years; 175 ± 9 cm; 72 ± 12 kg), were recruited to participate in this study. Individuals were excluded from participation if they had a history of lower limb injury, as well as any known neurological impairment, musculoskeletal issues or cardiovascular conditions.

### 2.2. Experimental design

#### 2.2.1. Three-dimensional foot scanning

The self-reported dominant foot of each participant was scanned under two load conditions, following procedures that have previously been shown to be reliable and repeatable [16]. For the first condition the foot was subjected to full bodyweight (fBW), which was achieved by having the participant support a mass equal to their body mass across their shoulders, while distributing the combined load (body mass and added mass) equally between both feet. For the second condition (mBW), as little load as possible was placed on the foot being scanned, which was achieved by having the participant seated, with the seat raised to the maximum height where the participant's heel would still be in contact with the surface of the scanner. All scans were captured using a FootIn3D scanner (Elinvision, Lithuania; reported accuracy of 0.3 mm) with a 1.4 mm mesh resolution.

#### 2.2.2. Plantar soft tissue stiffness

**Figure 1.**
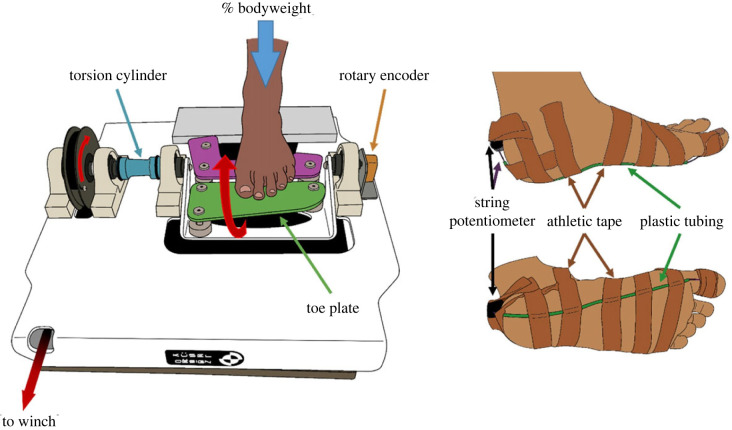
Left: toe extension rotation device used to extend the metatarsophalangeal joints while measuring degree of extension (rotary encoder) and moments (torsion cylinder). Right: position and attachment of the string potentiometer used to measure plantar soft tissue deformation. The string potentiometer's wire was fed through plastic tubing, which was attached to the plantar aspect of the foot using athletic tape.

A custom-built manual winch-driven device (figure 1) was used to extend the MTP joint from 0° (big toe horizontal and flat on the surface) to 45°, while the foot was subjected to 10% (= mBW) and 100% (= fBW) of the participant's body mass. Three MTP joint rotations were performed for each load condition. Foot loading was achieved in a similar manner as during the scans. In this case, the load placed on the foot was controlled using a force plate (OR6-7-1000, AMTI, USA) onto which the toe extension rotation device (TERD) was mounted. A rotary encoder (HEDL-5600-A06, Avago Technologies, USA), attached to one end of the TERD's crank shaft, was used to measure the degree of MTP joint extension. Meanwhile, two strain gauges (187UV, Micro Measurements, USA), mounted diametrically onto the torsion cylinder in a full Wheatstone bridge configuration, were used to measure the MTP joint moment. Lastly, a string potentiometer (173-0241-L2N, Firstmark Controls, USA) threaded through a tube that was attached with adhesive tape to the plantar aspect of the foot along the path of the medial band of the PA was used to measure plantar soft tissue deformation.

To determine the influence of active intrinsic foot muscle contractions on plantar soft tissue stiffness measures, a convenient subsample of 10 participants (five female, five male; 26 ± 3 years; 176 ± 8 cm; 71 ± 11 kg) returned on a later day to repeat the plantar soft tissue stiffness measurements (as described above), as well as with a tibial nerve block to prevent intrinsic foot muscle activation. A detailed description of the procedures, results and discussion thereof can be found in the supplementary material. Briefly, there were no differences in PA stiffness with and without the nerve block under mBW, and only a small decrease with the nerve block under fBW. From these results, it appears appropriate to consider our measures of plantar soft tissue stiffness as representative of PA stiffness and will thus be referred to as such.

### 2.3. Data processing and analysis

#### 2.3.1. Foot shape and deformation

The random order and variable number of vertices of the foot scans were addressed by elastically matching a reference scan to all other scans; thereby creating a group of uniquely shaped three-dimensional foot meshes with corresponding vertices. The registration algorithm used to establish the vertex correspondence consisted of an iterative affine transformation and an elastic deformation, as proposed by Danckaers *et al*. [39]. Any remaining differences in spatial alignment and scaling were removed with a generalized Procrustes analysis, followed by a principal component analysis (PCA) to determine the major modes of shape variation, or principal components (PC), within the group of foot scans. Lastly, so-called PC scores were assigned to each foot in order to place it within the distribution of shapes described by each PC.

Foot shape deformation between the mBW and fBW conditions was quantified by determining the Euclidean vectors joining each pair of corresponding vertices from scans of the same foot captured under the two load conditions [16]. As with foot shape, the major modes of deformation variation, as well as the place of each foot within the distribution of variations (PC scores), were determined using a PCA.

#### 2.3.2. Plantar aponeurosis stiffness

Plantar aponeurosis stiffness between the 0° and 45° of MTP joint extension was defined as in equation (2.1) [38,40],

2.1 $$\begin{array}{c} k=\;\frac{F}{\delta }, \end{array}$$

where *k* is the stiffness of the PA, *F* the force applied to the PA (i.e. the difference in force measured between the start and end of the rotation) and *δ* the displacement, or deformation, produced by the applied force. However, since PA force was not measured directly, it was defined as in equation (2.2),

2.2 $$\begin{array}{c} F=\;\frac{\tau }{r}, \end{array}$$

where *F* is the force applied to the PA, *τ* the torque measured about the MTP joint and *r* the moment arm of the MTP joint, which was obtained from fBW foot scans. To account for anthropometric differences between participants, PA force was normalized to body mass. A single stiffness value was calculated for each of the three trials per load condition and their mean value used for the subsequent statistical analyses.

### 2.4. Statistical analyses

Pearson's correlations were used to determine whether any of the mBW foot shape PCs were related to the foot deformation PCs. Paired *t*-tests were used to detect load-dependent differences in PA deformation, and bodyweight normalized PA force and stiffness.

Mixed forward and backward stepwise multiple linear regression analyses were performed in SPSS (v. 27, IBM, USA) to determine whether variations in foot shape, deformation, as well as their combination, explained variations in PA stiffness. To limit the number of predictor variables, only the scores from PCs accounting for 5% or more of the total variation, as well as the first PC below the 5% threshold, were used. For both foot shape and deformation this resulted in the inclusion of the first six PCs. These PCs accounted for 68% and 67% of the overall variation in foot shape and deformation, respectively (table 1).

**Table 1.** Absolute and cumulative percentage of total variance explained by the principal components (PC) of shape models of feet bearing minimal (mBW) and full bodyweight (fBW), as well as the deformation model between these two loads (mBW-fBW).

	mBW		fBW		mBW-fBW	
	absolute %	cumulative %	absolute %	cumulative %	absolute %	cumulative %
PC1	23.85	23.85	27.99	27.99	26.27	26.27
PC2	15.20	39.06	13.37	41.36	14.16	40.42
PC3	8.83	47.89	9.33	50.69	9.38	49.80
PC4	7.32	55.21	7.53	58.22	7.22	57.02
PC5	6.04	61.25	5.66	63.88	5.81	62.83
PC6	4.64	65.89	4.14	68.02	4.27	67.10

## 3. Results

### 3.1. Foot shape and deformation

**Figure 2.**
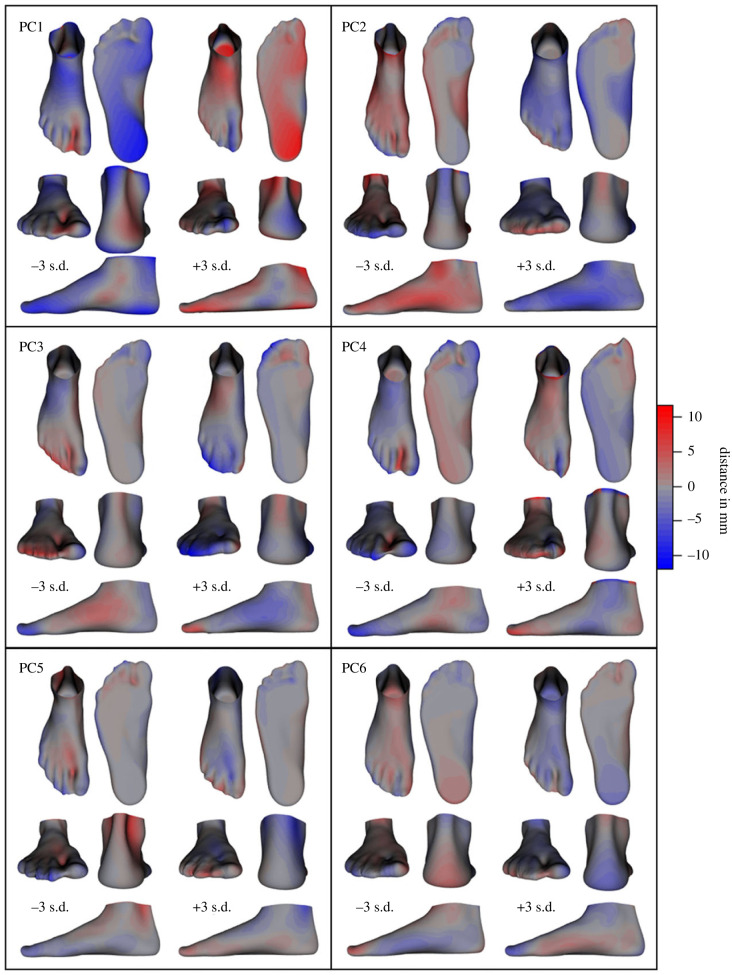
Principal components (PC) 1–6 of foot shape variations while bearing full bodyweight. The PCs are depicted as ± 3 s.d. of the mean fBW foot shape (figure 3). The red and blue heatmaps indicate the areas and magnitudes of differences to the mean foot. (Animations in the supplementary material help emphasize these shape differences for easier interpretation.)

The shape PCs examined in this investigation describe the same shape variances under both mBW and fBW conditions (figure 2), with respective amounts of total variance explained reported in table 1. Shape PC1 primarily describes differences in LA height, but also includes differences in heel shape, as well as forefoot and toe splay. PC2 is dominated by differences in overall foot width and TA curvature as seen across the dorsum of the foot (as illustrated in figure 5), whereas PC3 describes variations in forefoot abduction/adduction, as well as the length of the lesser toes. The remaining three PCs describe hallux splay, rearfoot alignment (PC4), Achilles tendon thickness, forefoot length (PC5), lesser toe alignment and hallux deviation (PC6).

**Figure 3.**
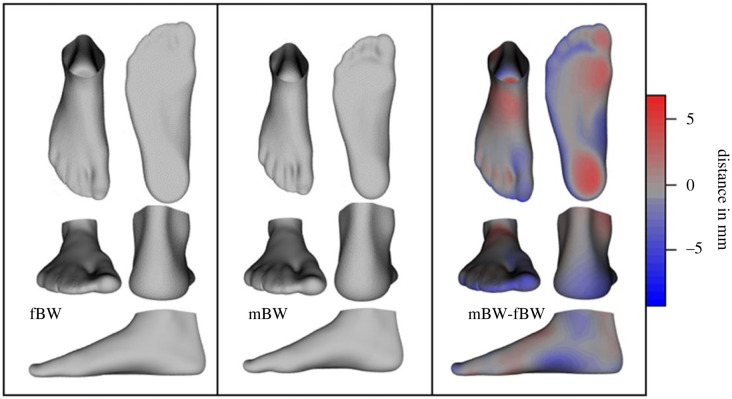
Mean foot shape while bearing full (fBW) and minimal bodyweight (mBW), as well as mean deformations that occur when the load is increased from mBW to fBW (mBW-fBW). The red and blue heatmaps on the mean deformed foot depict the areas and magnitude of differences between the mean mBW foot and the mean deformed foot. The foot shapes and deformations in this figure are the reference point from which the shape and deformation variations in figures 2 and 4 deviate.

**Figure 4.**
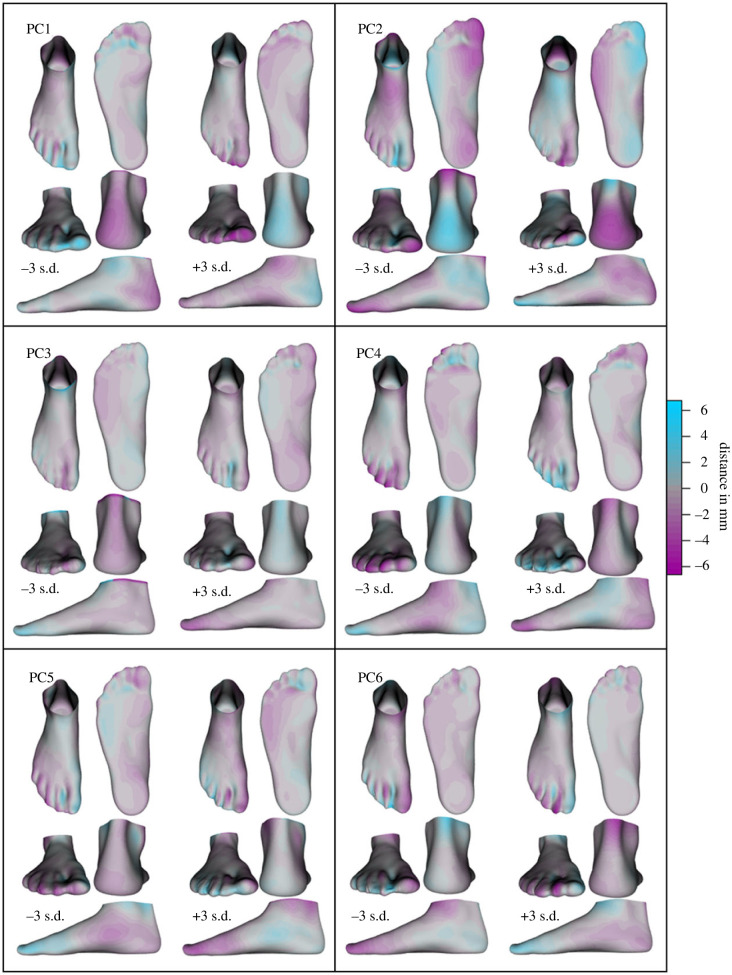
Principal components (PCs) 1–6 of foot deformations that occur when the load on a foot is increased from minimal (mBW) to full bodyweight (fBW). The PCs are depicted as the foot shape resulting from ± 3 s.d. of the deformations explained by each PC applied to the mean mBW foot (figure 3). The purple and teal heatmaps indicate the areas and magnitudes of differences to the mean deformed foot. (Animations in the supplementary material help emphasize these differences for easier interpretation.)

The mean foot deformation (figure 3) displays considerable compression of the plantar fat pads, flattening of the LA and hallux extension. The primary modes of deformation variance (PCs 1–3, approx. 50% explained variance, table 1) around this mean deformation appears to describe variations in hindfoot alignment, toe splay (PC1), LA compression, ankle rotation (PC2) and deviations of the hallux and lesser toes (PC3; figure 4). The midfoot also appears to be involved in the main deformation PCs through variations outside the sagittal plane, such as medial bulging due to forefoot alignment in the frontal and transverse planes (PCs 4 and 5; figure 4).

Though all mBW shape PCs correlate significantly with at least one deformation PC, all correlations were classified as weak to moderate (*r* < |0.5|, table 2). The fBW shape PCs also all correlate significantly, and with similar strength, with at least one deformation PC. Also noteworthy is that the first two mBW PCs do not correlate with the first two deformation PCs, whereas the first fBW PC does. Moreover, one of these correlations can be classified as strong (*r* > |0.5|, table 2).

**Table 2.** Pearson's correlation coefficients (*r*) between the first six principal components (PC) of the foot shape models for feet supporting minimal weight (mBW) or full bodyweight (fBW), and the foot deformation model (mBW-fBW). * and ** denote significant correlations (*p* < 0.05 and *p* < 0.01, respectively).

		deformation (mBW-fBW)					
	shape	PC1	PC2	PC3	PC4	PC5	PC6
mBW	PC1	0.081	0.170	0.148	0.030	0.346*	0.202*
	PC2	−0.082	0.055	0.237*	0.005	−0.455*	0.056
	PC3	0.117	−0.077	0.136	0.293*	−0.140	0.119
	PC4	**−0**.287*	**−0**.318*	−0.033	0.165	−0.128	−0.225*
	PC5	0.081	0.080	0.116	0.071	**0**.237*	−0.256*
	PC6	0.062	**−0**.203*	−0.029	−0.033	−0.105	0.088
fBW	PC1	**0**.306**	**0**.550**	0.022	−0.025	**0**.258**	0.177
	PC2	0.010	0.173	−0.066	−0.059	**0**.285**	−0.186
	PC3	−0.159	−0.054	0.132	−0.168	**−0**.264**	0.231*
	PC4	0.160	0.176	−0.330**	−0.121	−0.174	0.136
	PC5	−0.023	**−0**.213*	0.031	−0.176	−0.003	0.106
	PC6	−0.196	0.085	0.313**	−0.094	−0.045	−0.267**

### 3.2. Foot shape, deformation and plantar aponeurosis stiffness

Larger PA stiffness values were observed in fBW compared with mBW (*p* < 0.001). The load-dependent difference in PA stiffness is a result of increased PA force (*p* < 0.001), as PA deformation remained largely unchanged (*p* = 0.059) (table 3). The large variance in PA stiffness for both load conditions seems to be primarily a result of the large variance in PA force.

**Table 3.** Plantar aponeurosis force, stiffness (absolute and normalized to bodyweight) and deformation values for minimal (mBW) and full bodyweight (fBW) bearing. Values are presented as group means ± s.d.

	absolute		normalized		deformation (mm)
	force (N)	stiffness (N mm^−1^)	force (N kg^−1^)	stiffness (N kg^−1^ mm^−1^)	
mBW	82.63 ± 52.70	8.41 ± 6.02	1.18 ± 0.75	0.12 ± 0.09	10.28 ± 1.97
fBW	246.67 ± 147.87	24.17 ± 16.18	3.58 ± 2.27	0.35 ± 0.24	10.76 ± 2.15

Only a small amount of the variability in PA stiffness measured under both load conditions can be explained by their corresponding foot shape models. For mBW, a combination of PC3 and 4 (in order of decreasing contribution) explained 9.8% of the variability in PA stiffness (*F*_2,88_ = 5.911, *p* = 0.004). In fBW, PC3, 2 and 1 combined explained up to 14.3% of the variability in PA stiffness (*F*_3,86_ = 5.934, *p* = 0.001).

The deformations between the two load conditions can explain just under 11% of the variability in PA stiffness measured under each load condition (mBW: *F*_1,89_ = 8.526, *p* = 0.004, *r*^2^ = 0.108; fBW: *F*_1,88_ = 11.623, *p* = 0.001, *r*^2^ = 0.107). The deformations related to both mBW (PC5 and 6) and fBW PA stiffness (PC5) describe differences in forefoot abduction/adduction with medial bulging of the navicular tuberosity and extension of the hallux and lesser toes (figure 4).

Combining shape and deformation data to predict PA stiffness resulted in the same predictive capacity for fBW PA stiffness as when using deformation only (*F*_1,88_ = 11.623, *p* = 0.001, *r*^2^ = 0.107). Combining shape and deformation slightly increased the predictive capacity for mBW PA stiffness (*F*_4,86_ = 5.837, *p* < 0.001, *r*^2^ = 0.177) through a combination of the same shape (PC3 and 4) and deformation (PC5 and 6) variations previously identified as relevant to PA stiffness.

## 4. Discussion

Applying a novel approach to quantify foot shape and deformation, we have shown that differences in unloaded human foot shape are poor predictors of differences in foot deformation. Differences in loaded foot shape, in turn, are good predictors of some deformations and poor to moderate predictors of others. The strongest correlation being between descriptors of LA shape and flattening, which can be considered a surrogate measure of LA stiffness, since larger positive PC scores equate to greater compression and larger negative PC scores to reduced compression, despite the same increment in load. Most importantly, we show that variations in foot shape and deformation are poorly related to variations in our proxy of PA stiffness. Consistent with recent findings [19,41], our results suggest that long-held assumptions about human foot function that rely on close coupling between the shape, stiffness and the mechanical properties of the plantar aponeurosis, are overly simplistic and need to be re-evaluated.

### 4.1. Foot shape

**Figure 5.**
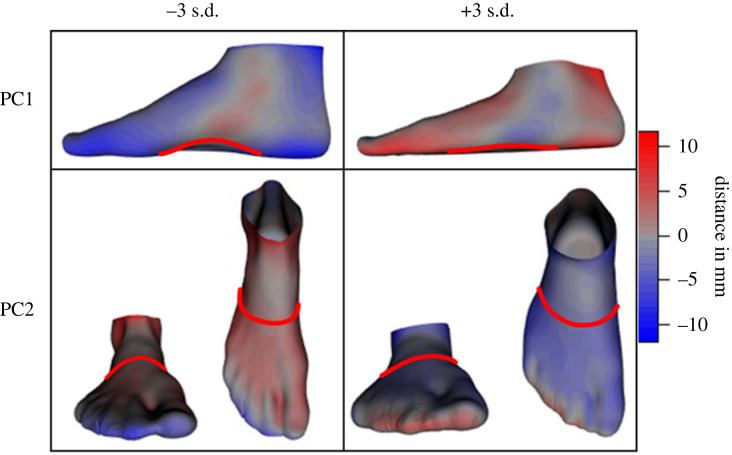
Variations in longitudinal and transverse arch shapes as described by the first and second principal components (PC1 and PC2, respectively) of the foot shape model while bearing full bodyweight. Foot shapes are depicted as the mean ± 3 s.d. of the respective PCs. Colour coding indicating the difference to mean foot shape and red lines are added to help emphasize these variations.

The primary source of shape variation within our sample of healthy human feet was related to the LA (PC1) and TA (PC2; figure 5), accounting for up to 28% and 15% of the variance (table 1) in the dataset, respectively. The LA and TA are uniquely human [1] and are believed to have evolved to stiffen the foot, allowing it to behave as a lever to propel the body forward during bipedal locomotion [22,34,42]. However, despite the apparent uniqueness and functional importance, the morphology of the LA and TA varies widely across healthy human feet, as observed here and in other recent studies using similar shape modelling approaches [14–16]. The substantial variation in the morphology of the LA and TA indicates that the shape of these structures may not be tightly constrained features of the human foot, and suggests that the shape of the LA and TA may not be as important for human locomotion as widely believed [1]. For example, if a specific arched shape was required to provide stiffness for propulsion, or to store and return a given amount of mechanical energy, it could be expected that this shape feature would be tightly constrained across the human species, to deliver the required function [43]. However, as proposed by McClymont *et al*. [44], given the large degrees of freedom provided by the multiple articulations within our feet, it is likely that the required functions can be obtained with a wide variety of shape configurations.

Developmental plasticity may also explain some of the variance in general foot shape, deformation and PA stiffness within our dataset. For example, the level of physical activity and the footwear used across our lifespan (or even over a relatively short period of time) can influence the width and height, as well as the volume of muscle within the arch of our feet [45,46]. However, given that our group of participants were healthy, active adults who had habitually worn shoes for most of their lives, we believe it is unlikely that this would be a major contributor to the shape variations seen here.

### 4.2. Foot deformation

Our foot deformation model provides unique insight into the three-dimensional shape changes that occur in healthy human feet, under load. An important aspect of our foot deformation model is the mean foot deformation, which represents the average three-dimensional foot deformation that occurs under load, across all of our dataset (figure 3). The variance in shape deformation away from this mean is explained by the shape deformation PCs. An interesting observation regarding these deformations is that, despite the variations described by PC2, substantial LA flattening is also included in the mean foot deformation. The fact that LA compression is common in all feet supports the recent studies from our laboratory that have revealed the importance of LA mobility in facilitating versatility in human foot function, allowing the foot to actively transition between the mechanical functions of a spring, a damper and a motor during a wide variety of locomotor tasks [47–51]. Also of note are the multi-planar variations in midfoot deformations [7]. Though sagittal plane LA compression displays a large amount of variability (approx. 14% of total variability), some form of midfoot motion is included in all but one of the first six PCs.

### 4.3. Foot shape and deformation

Here we have shown that the shape of an unloaded foot is a poor predictor of the way that foot will deform when loaded, whereas certain shape characteristics of a loaded foot can predict some of these deformations. The strongest relationship between foot shape and deformation was found to be between PC1 of the loaded foot shape model (approx. 28% explained variance), which primarily explains variance in LA shape, and PC2 of the deformation model (approx. 40% explained variance, table 1), which includes variations in sagittal plane LA flattening. The observed relationship between LA shape and compression provides further support for the long-standing hypothesis that the arched shape of human feet is a determinant of their longitudinal stiffness [34,35,52]. However, our results also show that LA compression is neither the only nor the most variable deformation associated with LA shape. Both rearfoot and forefoot alignment (mBW-fBW PCs 1 and 5, respectively) are also associated with the primary mode of shape variance. Especially forefoot alignment seems to be a quite common motion during loading as it was found to be related to each of the first three modes of shape variation. Together, these findings highlight the complex three-dimensional nature of foot deformation. As such, simple measures of LA flattening may not provide an accurate description of the multi-faceted relationships between the shape of a foot and its stiffness.

The TA may also play an important role in delivering stiffness to the human foot, as recently highlighted by Venkadesan *et al*. [22]. Using a series of mathematical and physical models, alongside a small number of cadaveric loading experiments, Venkadesan and colleagues provide a clear explanation for how the curvature (shape) of the TA might provide stiffness to the human foot. The underlying premise for this model was based on comparative observations between human and non-human primate feet and analysis of fossil records. A limitation to this eloquent series of modelling experiments is a lack of *in vivo* human foot data that tests the mechanism across a spectrum of healthy human feet, which would have allowed the authors to account for the variability in TA curvature and foot stiffness that is observed across a multitude of feet with varying shape and stiffness configurations. Our *in vivo* approach has allowed us to explore this relationship and indirectly test the robustness of the proposed mechanism across a wide variety of healthy human feet. Based on our static deformations, it appears that TA shape (fBW PC2; figure 5) is neither related to LA shape (fBW PC1; figure 5) nor flattening (mbW-fBW PC2) but is instead associated with LA deformation through frontal plane motion at the midfoot (mBW-fBW PC5). Nevertheless, further evaluation of the mechanism proposed by Venkadesan and colleagues based on dynamic measures of foot stiffness during locomotion may help to further parse out the relationship between TA shape and LA stiffness.

### 4.4. Foot shape, deformation and plantar aponeurosis stiffness

The PA is a central structure in mechanical models of human foot function [1,17,26] and is thought to be an important determinant of both foot shape and stiffness [3,19,37,53–56]. By contrast to these findings, our results indicate that foot shape and deformation are not as closely linked to PA stiffness as previously assumed. We found external foot shape features to be poor predictors of PA stiffness, regardless of the load placed on the foot. Unlike the 52% change in foot posture index (FPI) scores explained by PA thickness [37], foot shape features only accounted for approximately 10–14% of change in PA stiffness. Furthermore, LA shape (as described by fBW PC1) only contributes toward increasing the predictive capacity of PA stiffness once toe position (PC3), TA curvature and foot width (PC2) have been accounted for. In the mBW condition, neither of the two predictor shape PCs involve the LA. Both of these relationships have a shared limitation in that the methods used to quantify foot form do so under static conditions, while the interaction between LA and PA are primarily dynamic in nature. Nevertheless, our results also indicate that foot deformation, a dynamic measure of foot shape and a surrogate for foot stiffness, is a comparably poor predictor of PA stiffness across individuals. Only the combination of foot shape and deformation resulted in a better, but still limited, predictive capacity (approx. 18%). These same or better results were attained by taking into account PCs that describe midfoot motions in the frontal plane (mBW-fBW PC5) and lesser toe position (PC6), while disregarding PCs that describe variations in LA shape, which further strengthen the argument that the relationship between how the foot handles load and the characteristics of its constituent parts are not as straightforward as initially thought.

There are two further, alternative explanations for the poor relationships between foot shape and deformation characteristics, and PA stiffness. Firstly, the conditions under which we are observing foot shape, deformations, and the behaviour of the PA may be too different to compare. The deformations were measured as a consequence of increased load while the foot was flat on the ground. When running, this occurs during approximately 20–60% of the stance phase, throughout which the PA has been shown to elongate [19]. Meanwhile, PA stiffness was measured by dorsiflexing the MTP joints of an already loaded foot, which is closer to the push-off phase (approx. 60–80%) during which the PA acts quasi-isometrically and shortens [19]. Secondly, the high variability in both external foot shape and deformation includes factors that do not affect LA dynamics, such as the plantar fat pads (included in most deformation PCs) and the shape of the heel and toes (included in most shape PCs). By introducing additional noise in the shape and deformation data, variations in fat pad deformations and heel and toe shape might obscure any true relationship between LA shape, deformation and PA stiffness. Thus, shape representations of the foot bones, which have been shown to be closely related to midfoot mobility [23], and changes in their configuration due to loading might be better predictors of PA stiffness. Contrary to this argument, however, bone geometry and radiographic images have been shown to be related to foot type classifications based on external foot shape [57], and radiographic measurements of foot structure were only able to account for 35% of the variation in plantar pressure [58]. While the former does not necessarily mean that the inverse is also true (i.e. that external foot shape is a sufficiently accurate representation of internal structure), the latter does indicate that, as suggested here, internal structure and external function of the foot are not as closely linked as often assumed.

Our results support the general consensus that foot form and PA properties are somehow linked but challenge the previously assumed strength of this link. The weak relationships between static foot shape and PA stiffness support the recent finding and suggestions that the PA is not a major determinant of LA form [53,59], while both the magnitudes and nature of relationships between foot shape and deformation, as well as foot deformation and PA stiffness, support the recent discovery that the dynamic interactions between LA and PA are more complex than the originally proposed windlass and spring-like mechanisms [19,60]. In the light of these revised LA and PA interactions, and to better understand how modern human feet function, it remains to be determined how the wide variations in PA stiffness affect LA dynamics during locomotion. Furthermore, the weak relationships between foot shape, deformation and PA stiffness also highlight the complexity of the structures of the foot and their interactions. Hence, simple comparisons between the characteristics of isolated foot structures, as is common in palaeoanthropological studies, may yield oversimplified and misleading results. Determining whether and to what extent the relationships observed here translate to relationships between foot shape, deformation and energetics during locomotion could provide valuable context when attempting to infer gait mechanics from foot morphology, as is necessary when studying the evolution of modern human bipedalism.

## 5. Limitations

There are a few limitations regarding our methods that should be considered. Unlike the *ex vivo* studies, our measures of PA deformation and force are indirect and thus susceptible to various sources of random and systematic error. First is the inevitable variability in placement of the string potentiometer that may have affected the measurements of PA deformation. The same can be said for the lack of control of the centre of pressure under the foot during MTP joint extensions and MTP joint flexion torques. Lastly, measuring MTP joint moment arms from foot scans may also have affected the resulting PA forces. Nonetheless, the coincidence between our and previously reported *ex vivo* values [61,62] supports the validity of our measurements, while the limited sample sizes used previously make it hard to gauge whether the currently observed variability is due to error or not (see electronic supplementary material). Though further research (ideally using direct *in vivo* measurements) would be required to guarantee the validity and reliability of the results presented here, they still offer valuable insight into the wide range of PA stiffness values that can be expected within a healthy adult population.

## 6. Conclusion

The arched configuration of our feet has correctly been identified as an important component of their external shape as well as an important determinant of their longitudinal stiffness. While the shape of a loaded foot is a good predictor of how that foot will deform when encumbered with load, the same cannot be said for the shape of an unloaded foot. Furthermore, wide variation in PA stiffness, as well as its poor relationship to both foot shape and deformation (especially that of the arches) emphasize the structural and functional complexity of the foot, which seems to provide the necessary leeway to achieve comparable functional outcomes despite wide variations in structure. Thus, this study adds to the accumulating evidence indicating that, despite having some merit, simplified models and isolated measures cannot accurately describe the complex relationship between shape and mechanical function of modern human feet. To be able to accurately describe this relationship, and improve the conclusions and inferences based thereon, a more elaborate approach seems necessary.

## Data Availability

The data for this manuscript has been published in The University of Queensland Research Data Manager and can be accessed via the following link: https://doi.org/10.48610/a559c92 [63]. Additional information and animated figures are provided in electronic supplementary material [64].
